# BasS/BasR Two-Component System Affects the Sensitivity of *Escherichia coli* to Plantaricin BM-1 by Regulating the Tricarboxylic Acid Cycle

**DOI:** 10.3389/fmicb.2022.874789

**Published:** 2022-04-14

**Authors:** Yifei Liu, Yawen Wang, Xinyue Chen, Junhua Jin, Hui Liu, Yanling Hao, Hongxing Zhang, Yuanhong Xie

**Affiliations:** ^1^Beijing Laboratory of Food Quality and Safety, Beijing Key Laboratory of Agricultural Product Detection and Control of Spoilage Organisms and Pesticide Residue, College of Food Science and Engineering, Beijing University of Agriculture, Beijing, China; ^2^Beijing Advanced Innovation Center for Food Nutrition and Human Health, College of Food Science and Nutritional Engineering, China Agricultural University, Beijing, China

**Keywords:** bacteriocins, BasS/BasR two-component system, proteome, tricarboxylic acid cycle, *Escherichia coli*

## Abstract

Plantaricin BM-1, a class IIa bacteriocin produced by *Lactiplantibacillus plantarum* BM-1, exhibits significant antibacterial activity against many gram-positive and gram-negative bacteria. However, the mechanism underlying the action of class IIa bacteriocins against gram-negative bacteria remains to be explored. This study aimed to investigate the role of the BasS/BasR two-component system (TCS) in *Escherichia coli* (*E. coli*) K12 response to plantaricin BM-1. The IC_50_ values for plantaricin BM-1 in *E. coli* K12, *basS* mutant (*E. coli* JW4073), and *basR* mutant (*E. coli* JW4074) strains were found to be 10.85, 8.94, and 7.62 mg/mL, respectively. Growth curve experiments showed that mutations in the BasS/BasR TCS led to an increase in the sensitivity of *E. coli* K12 to plantaricin BM-1 and that after gene complementation, the complemented mutant strain regained its original sensitivity. Proteomic analysis showed that 100 and 26 proteins were upregulated and 62 and 58 proteins were downregulated in *E. coli* JW4073 and *E. coli* JW4074, respectively. These differential proteins, which exhibited different molecular functions and participated in different molecular pathways, were mainly concentrated in the cytoplasm. More specifically, mutations in *basS* and *basR* were found to affect the synthesis and metabolism of many substances in *E. coli*, including many important amino acids and enzymes involved in cellular activities. In addition, 14 proteins, including 8 proteins involved in the tricarboxylic acid (TCA) cycle, were found to be downregulated in both *E. coli* JW4073 and *E. coli* JW4074. Growth curve experiments showed that the deletion of these proteins could increase the sensitivity of *E. coli* to plantaricin BM-1. Therefore, we speculate that TCA pathway regulation may be an important mechanism by which the BasS/BasR TCS regulates the sensitivity of *E. coli* to plantaricin BM-1. This finding will facilitate the determination of the mechanism underlying the action of class IIa bacteriocins against gram-negative bacteria.

## Introduction

Bacteriocins are 20–60 amino acid-long ribosomally synthesized and secreted antimicrobial peptides (AMP) capable of inhibiting both gram-negative and gram-positive food spoilage/pathogenic bacteria (Kumariya et al., 2019). Due their high potency and specificity, their effectiveness as inhibitors of pathogenic and spoilage microorganisms has been extensively investigated (Telhig et al., 2020). Thus, bacteriocins are widely considered to be promising antimicrobial agents that can be used for different purposes, including food preservation and the treatment of bacterial infections (Cotter et al., 2005; Hassan et al., 2012).

Class IIa bacteriocins, which are heat-stable, unmodified peptides with a conserved amino acid sequence (YGNGV) in their N-terminal domains, have received much attention due to their “generally recognized as safe (GRAS)” status, their high biological activity, and their excellent heat stability (Cui et al., 2012). Many lactic acid bacteria produce class IIa bacteriocins with anti-listerial activity (Kjos et al., 2011). The successful application of pediocin PA-1 as a bio-preservative in the food industry has promoted research into the mechanism of action of class IIa bacteriocins (Rodríguez et al., 2002). The target receptor for class IIa bacteriocins in gram-positive bacteria has been identified to be constituted of proteins of the sugar transporter mannose phosphotransferase system (Man-PTS). The Man-PTS consists of four subunits, IIA, IIB, IIC, and IID. Through gene deletion and complementation studies it has been shown that the membrane-located proteins, IIC and IID, together form the bacteriocin receptor, while the IIA and B subunits are dispensable for receptor function (Diep et al., 2007). However, evidence has shown that the Man-PTS is not the action site for class IIa bacteriocins in Gram-negative bacteria. Thus, the mechanism of action of class IIa bacteriocins in gram-negative bacteria is yet to be elucidated.

The two-component system (TCS) is a signal transduction pathway widely employed by prokaryotic and eukaryotic organisms. Typically, the TCS is composed of a sensor that monitors external signals and a response regulator (RR) that controls gene expression and other physiological activities, such as chemotaxis (West and Stock, 2001). Most sensors of the bacterial TCS are membrane-associated histidine kinases (HKs). The sensor autophosphorylates its conserved His residue in response to signals from the environment. The HK carboxyl-terminal cytoplasmic region, which is known as the transmitter domain, consists of an ATP-binding domain and an H box domain that contains the conserved His residue that undergoes autophosphorylation. Subsequently, the HK His-bound phosphoryl group is transferred onto a specific Asp residue on the cognate RR for its activation. In most cases, the activated RR induces the transcription of a set of genes which respond to external signals (Nagasawa et al., 1993). *E. coli* possesses several His-Asp phosphorelay signal transduction systems, each of which is involved in a different type of stress response and/or adaptation (Mizuno et al., 2003). The BasS/BasR TCS is an iron- and zinc-sensing transcription regulator in *E. coli* (Lee et al., 2005). Studies have shown that in *E. coli* K12, BasSR directly regulates a set of genes associated with metal-response-mediated membrane structure modification and the modulation of membrane functions, as well as genes associated with response to acidic and/or anaerobic growth conditions (Hagiwara et al., 2004; Ogasawara et al., 2012). In addition, the BasS/BasR TCS can induce the upregulation of genes related to biofilm formation in Avian pathogenic *E. coli* (APEC) (Yu et al., 2020).

*Lactiplantibacillus plantarum* BM-1, isolated from a traditional fermented meat product, was found to produce a new type IIa bacteriocin, plantaricin BM-1, which was shown to exhibit significant inhibitory activity against some foodborne bacteria, including *E. coli* (Zhang et al., 2013). In a previous study, we found that plantaricin BM-1 induces *E. coli* K12 cell rupture and depression by interacting with its cell membrane, thereby inhibiting its growth (Wang et al., 2020). The expression of the BasS and BasR was found to be upregulated by the deletion of *ybfA*, and this promoted cell membrane modifications in *E. coli* and protected the bacteria by reducing bacteriocin-induced damage (Chen et al., 2021). However, what role BasS/BasR TCS plays in the sensitivity of *E. coli* to bacteriocin is still unknown. In this study, we mainly investigated the regulatory role of BasS/BasR TCS in the sensitivity of *E. coli* to plantaricin BM-1. By studying the possible regulatory mechanism of BasS/BasR TCS, we have found BasS/BasR TCS may have regulatory functions regarding tricarboxylic acid (TCA).

## Materials and Methods

### Antimicrobial Protein Preparation

Plantaricin BM-1 was prepared according to the method reported in our previous study (Zhang et al., 2013). In brief, *Lactiplantibacillus plantarum* BM-1 was cultured in MRS broth at 37°C for 20 h. The supernatant of the fermentation broth was collected by centrifugation, and plantaricin BM-1 was purified by dialysis, desalting, and cation exchange. The purified plantaricin BM-1 was freeze-dried and stored at −80°C.

### Strains and Culture Conditions

The bacterial strains used in this study are listed in Table 1. *E. coli* strains were cultured in Luria-Bertani (LB) broth at 37°C with aeration at 180 rpm. *Lactobacillus plantarum* BM-1 was cultured in de Man, Rogosa, and Sharpe (MRS) broths at 37°C with aeration at 180 rpm.

**TABLE 1:** Strains used for the study.

Strains and plasmids	Characteristics	Source
*E. coli* K12	Wild-type *E. coli* strain BW25113	Laboratory preservation (Wang et al., 2020)
*E. coli* JW4073	*E. coli* BW25113 with *basS* deletion	Keith collection (Tomoya et al., 2006)
*E. coli* JW4074	*E. coli* BW25113 with *basR* deletion	Keith collection (Tomoya et al., 2006)
*L. plantarum* BM-1	*Lactobacillus plantarum* BM-1, producing plantaricin BM-1	Laboratory preservation (Zhang et al., 2013)
pKD46	Plasmid containing the lambda Red system, L-arabinose inducible	BioVector NTCC
*E. coli* ReJW4073	*E. coli* JW4073 with *basS* complemented	This study
*E. coli* ReJW4074	*E. coli* JW4074 with *basR* complemented	This study
*E. coli* JW3923	*E. coli* BW25113 with *pflD* deletion	Keith collection (Tomoya et al., 2006)
*E. coli* JW4084	*E. coli* BW25113 with *dcuB* deletion	Keith collection (Tomoya et al., 2006)
*E. coli* JW0616	*E. coli* BW25113 with *dcuC* deletion	Keith collection (Tomoya et al., 2006)
*E. coli* JW1604	*E. coli* BW25113 with *fumA* deletion	Keith collection (Tomoya et al., 2006)
*E. coli* JW2235	*E. coli* BW25113 with *glpA* deletion	Keith collection (Tomoya et al., 2006)
*E. coli* JW2236	*E. coli* BW25113 with *glpB* deletion	Keith collection (Tomoya et al., 2006)
*E. coli* JW2237	*E. coli* BW25113 with *glpC* deletion	Keith collection (Tomoya et al., 2006)

### Determination of Semi-Inhibitory Concentrations (IC_50_)

The concentration of the purified plantaricin BM-1 was determined using a NanoDrop 2000 spectrophotometer (Thermo Fisher Scientific, MA, United States). Then, double dilution solutions (55.32, 27.66, 13.83, 6.915, 3.4575, 1.72875, 0.864385, 0.4321875, and 0.21609375 mg/mL) were prepared using sterile water under sterile conditions. Equal amounts of the diluted solutions were added to the wells of 96-well plates (100 μL per well) according to the dilution gradient, and the same amount of sterile water was used as the negative control. *E. coli* at the logarithmic growth phase was collected and used to prepare a 10^4^ CFU/mL bacterial suspension. Then, 100 μl of the *E. coli* suspension was added to each well. After mixing, the plates were incubated at 37°C for 12 h, and the optical density (OD) at 600 nm (OD_600_) was determined using an ELISA plate reader (ELX808; BioTek, VT, United States). Each experiment was performed in triplicate. Based on the OD_600_ values, the *E. coli*-inhibitory rate of each sample was calculated. Then, Graphpad Prism 7.00 was used to calculate the IC_50_.

### Construction of *basS*-Complemented Strain (*Escherichia coli* ReJW4073) and *basR*-Complemented Strain (*Escherichia coli* ReJW4074)

*basS* complemented strain (*E. coli* ReJW4073) and *basR* complemented strain (*E. coli* ReJW4074) were constructed using the lambda Red homologous recombination method (Juhas and Ajioka, 2016). In brief, the pKD46 plasmid was transformed into competent mutant cells prepared with cold 0.1 mol/mL CaCl_2_, and the expression of the homologous recombinase in pKD46 was induced by adding 0.5 mg/mL L-arabinose to the LB broth and incubating at 30 °C. Then, competent mutant cells carrying the pKD46 plasmid were obtained. *basS* gene fragments were amplified from *E. coli* K12 using the primers, BasS-F (5′-3′): AGCGTGCTGGTGGTCAGCAGCTTTCTTTATATCTGGTTT GCCACGTACTGA and BasS-R (5′-3′): CTTACTCCTTT TGATTAACTTAGACATGCATTTTCTGCGCCGACCAATAT (homology underlined), while *basR* gene fragments were amplified from *E. coli* K12 using the primers, BasR-F (5′-3′): CGGCGCAGAAAATGCATCAGATTCAATTAGTTTTCCTCA TTCGCGACCAG and BasR-R (5′-3′): CGTTTGAACGTC CTCTCACTCACTTTGAAAATTCTGATTGTTGAAGACGA (homology underlined). Then, these fragments were transfected into the prepared competent *E. coli* JW4073 and *E. coli* JW4074 cells. Next, 900 μL of LB broth was added to the cells and incubated at 37°C for 1 h. Then, the cells were diluted with physiological saline, spread on LB agar, and incubated at 37°C for 12 h. A single colony was selected and verified using the BasS-F/R and BasR-F/R primers.

### Growth Curves for *Escherichia coli* K12, *Escherichia coli* JW4073, *Escherichia coli* JW4074, and the Compensatory Strains Treated With Plantaricin BM-1

Wild-type *E. coli* K12, *E. coli* JW4073, *E. coli* JW4074, *E. coli* ReJW4073, and *E. coli* ReJW4074 were cultured in LB broth at an initial concentration of 4.0 log_10_ CFU/mL with or without plantaricin BM-1 (0.5 × IC_50_ of *E. coli* K12) at 37 °C for 12 h. Samples of the bacterial solution were collected 2-hourly and transferred onto a plate for cell counting, and the mean values of the results of three experiments were plotted. All experiments were performed in triplicate.

### Proteomic Analysis

Quantitative proteomic analysis of wild-type *E. coli* K12 and mutant strains was carried out by Tandem mass tags (TMT) to screen for differentially expressed proteins between the three strains, and bioinformatic analysis was performed to compare differentially expressed proteins between the different groups. These analyses were performed by Shanghai Meiji Biotechnology Co., Ltd., and the specific steps were as follows:

*E. coli* K12, *E. coli* JW4073, and *E. coli* JW4074 strains were cultured in LB broth (at a final concentration of 10^4^ CFU/mL) at 37 °C for 12 h without plantaricin BM-1. After centrifugation, the pellet was collected and treated with protein lysis buffer (8 M urea + 1% sodium dodecyl sulfate, protease inhibitor) to obtain total proteins. The concentration of the extracted proteins was determined using the Pierce BCA protein assay kit (Thermo Fisher Scientific, MA, United States). Demethylation was carried out by the addition of 100 mM (2-carboxyethyl) phosphine and 40 mM iodoacetamide into the total protein extract. Then, the proteins were hydrolyzed using trypsin at a 1:50 ratio. Hydrolytic peptides were labeled using a TMT labeling kit (Thermo Fisher Scientific, MA, United States), and then subjected to liquid chromatography-tandem mass spectrometry (LC-MS/MS). Three biological replicates were prepared for each sample.

High-pH liquid-phase TMT-labeled peptide separation was performed using a reversed phase liquid chromatographic system (Thermo Scientific Vanquish Flex, Thermo Fisher Scientific, MA, United States) equipped with a reversed-phase C18 column (ACQUITY UPLC BEH C18 Column, 1.7 μm, 2.1 mm × 150 mm, Waters, United States). Peptide elution was monitored at 214 nm; after 5 min, the eluted peptides were collected every minute, pooled into 10 fractions, and then lyophilized. The dried fractions obtained from the high-pH reverse-phase separation process were run on a Q Exactive mass spectrometer (Thermo Fisher Scientific, MA, United States) equipped with a C18 column (75 μm × 25 cm, Thermo Fisher Scientific, MA, United States) for their identification and quantification using previously reported identification parameters.

Liquid chromatography-tandem mass spectrometry data were matched using the Proteome Discoverer TM version 2.2 software, and searched on the uniprot-*E. coli* (strain K12) [83333]-4353s-20190412 database, with a ≤0.01 peptide false discovery rate. Only proteins containing at least one unique peptide were quantified. Subsequently, differentially expressed proteins were identified based on fold-change >1.2 or <0.83 between treatments and *p* < 0.05. Kyoto Encyclopedia of Genes and Genomes (KEGG) pathway enrichment analysis^1^ was used to identify the functional subclasses and metabolic pathways of the differentially expressed proteins.

### Real-Time Quantitative PCR

Wild-type *E. coli* K12 and mutant *E. coli* JW4073 and *E. coli* JW4074 strains were cultured in liquid LB broth at 37°C for 12 h at an initial concentration of 4.0 log_10_ CFU/mL. Total bacterial RNA was extracted using a bacterial RNA extraction kit (Vazyme Biotech, Nanjing, China). Then, Real time quantitative PCR (RT-qPCR) was performed using a Hiscript II One Step qRT-PCR SYBR Green kit (Vazyme Biotech, Nanjing, China). Using 1 μg RNA as a template, 10 μL of 2 × One step SYBR Green mixture, 1 μL of one-step SYBR Green enzyme mixture, 0.4 μL of 50 × Rox reference dye, 0.4 μL of gene specific primer forward (10 μM), and 0.4 μL gene specific primer reverse (10 μM) were added to a ribonuclease-free centrifuge tube, and RNase free ddH_2_O was added to make up the volume to 20 μL. The RT-qPCR reaction cycling conditions were as follows; 50°C for 15 min, 95°C for 30 s, 95°C for 10 s, 60°C for 30 s, and 40 cycles. The primers shown in Table 2 were used for the PCR reaction. Using the wild-type *E. coli* K12 gene expression level as the internal reference factor, relative gene expression in mutant *E. coli* JW4073 and *E. coli* JW4074 was calculated by the 2^–ΔΔCT^ method.

**TABLE 2:** Primers for RT-qPCR.

Primers name	Primer sequence (5′-3′)
16S-F	ACCCTTATCCTTTGTTGCC
16S-R	TCTTTGTATGCGCCATTGTA
GlpA-F	GACCTATCGGCTGATGGCTGAATG
GlpA-R	GCAGGCAGGGAGATGACTTTACG
GlpB-F	TCACAGGCAGGGCAAACCATTG
GlpB-R	GTAAAGCACTGACGGCACAAACAC
GlpC-F	TAAAGCACGCAAACAGGCAATTACG
GlpC-R	GTACAGGTTGAGGAGGTGGCAATC

### Analysis of the Sensitivity of BasS/BasR Two-Component System-Regulated Tricarboxylic Acid -Related Genes to Plantaricin BM-1

*E. coli* JW3923, *E. coli* JW4084, *E. coli* JW0616, *E. coli* JW1604, *E. coli* JW2235, *E. coli* JW2236, and *E. coli* JW2237 strains were cultured in LB broth at 37 °C for 12 h at an initial concentration of 4.0 log_10_ CFU/mL, with or without plantaricin BM-1 (1 × IC_50_ of *E. coli* K12). Samples of the bacterial suspension were collected every 2 h and transferred onto a plate for cell quantification, and the mean values of the results of three experiments were plotted. All experiments were performed in triplicate.

### Statistical Analysis

All experiments were performed in triplicate, and data are presented as the mean ± SD. Analysis of variance was used to compare viable cell counts between the growth curves of *E. coli* treated with and without plantaricin BM-1 at a significance level of 0.05.

## Results

### Determination of Semi-Inhibitory Concentrations

After culturing the bacterial strains in various plantaricin BM-1 concentrations at 37°C for 12 h, the OD_600_ of each bacterial suspension was determined. The IC_50_ of plantaricin BM-1 in *E. coli* K12, *E. coli* JW4073, and *E. coli* JW4074 strains were determined to be 10.85, 8.94, and 7.62 mg/mL, respectively.

### Construction of the *basS*-Complemented *Escherichia coli* JW4073 and the *basR*-Complemented *Escherichia coli* JW4074 Mutants

The 1,092 bp *basS* gene fragment and the 669 bp *basR* gene were amplified from the *E. coli* K12 genome by PCR using the BasS-F/R and BasR-F/R primer pairs, and were subsequently used to replace the kanamycin resistance gene in *E. coli* JW4073 and *E. coli* JW4074 via red homologous recombination. Genomic DNA was extracted from *E. coli* and its corresponding complement strain, and PCR was performed on both genomes using the BasS-F/R and BasR-F/R primer pairs to confirm successful mutant construction.

**FIGURE 1:**
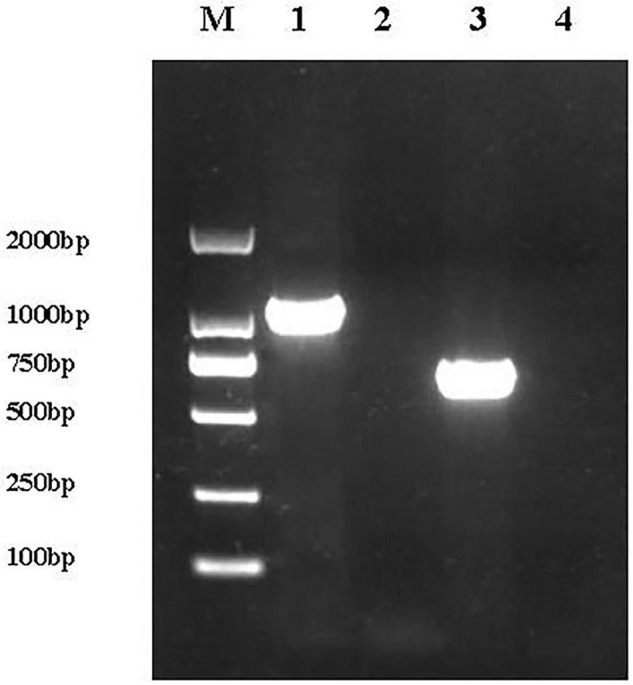
Verification of *E. coli* ReJW4073 and *E. coli* ReJW4074 construction. PCR products were detected by 2% agarose gel electrophoresis. Lane M represents the 2,000 bp DNA marker, lane 1 contains the PCR product from *E. coli* ReJW4073, lane 2 contains the PCR product from *E. coli* JW4073, lane 3 contains the PCR product from *E. coli* ReJW4074, and lane 4 contains the PCR product from *E. coli* JW4074.

A 1,092 bp product was amplified from the complemented *E. coli* ReJW4073 strain, but no amplified band was detected for *E. coli* JW4073; in addition, a 669 bp product was amplified from the complemented *E. coli* ReJW4074 strain, but no amplified band was detected for *E. coli* JW4074 (Figure 1). Sequencing results showed that the sequences of the amplified gene fragments from *E. coli* ReJW4073 and *E. coli* ReJW4074 were the same as those of the *basS* and *basR* gene fragments of *E. coli* K12, respectively, indicating that *E. coli* ReJW4073 and *E. coli* ReJW4074 were successfully constructed.

### Growth Curves for *Escherichia coli* K12, *Escherichia coli* JW4073, *Escherichia coli* JW4074, *Escherichia coli* ReJW4073, and *Escherichia coli* ReJWW4074 Under Plantaricin BM-1 Treatment

**FIGURE 2:**
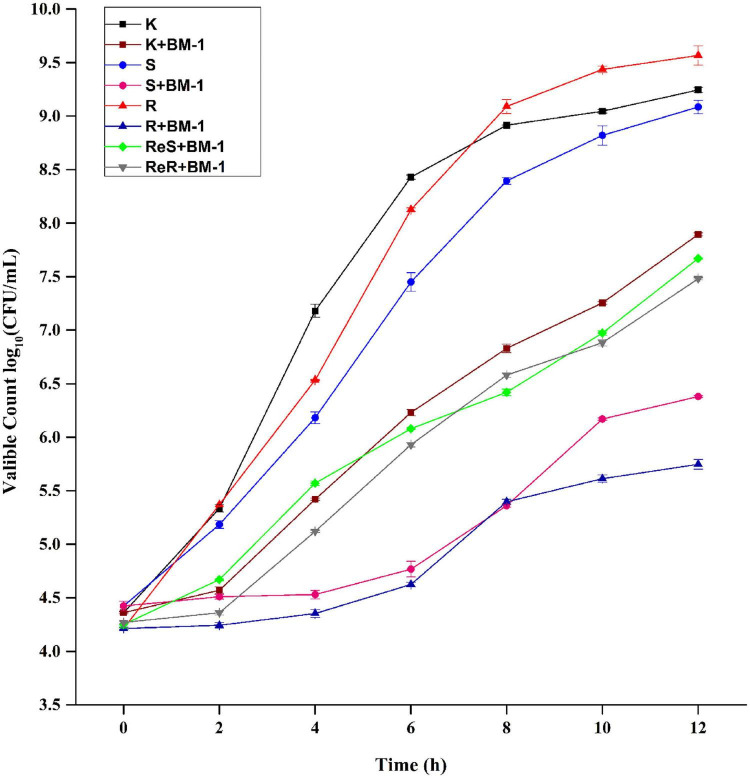
Effects of plantaricin BM-1 on the growth of wild type *E. coli* K12 (K) and mutant *E. coli* JW4073 (S), *E. coli* JW4074 (R), *E. coli* ReJW4073 (ReS), and *E. coli* ReJW4074 (ReR).

The effects of plantaricin BM-1 on the growth of *E. coli* K12, *E. coli* JW4073, *E. coli* JW4074 *E. coli* ReJW4073, and *E. coli* ReJWW4074 strains were assessed through the generation of standard growth curves (Figure 2). In the absence of plantaricin BM-1, all three strains showed similar exponential growth. *E. coli* K12, *E. coli* JW4073, and *E. coli* JW4074 cell counts at 12 h were 9.3, 9.5, and 9.6 log_10_ CFU/mL, respectively. *E. coli* JW4073 and *E. coli* JW4074 had similar growth conditions, and their curves almost overlapped, probably due to the fact that mutations in *BasS* and *BasR* induce similar effects. Although the three strains eventually reached a similar peak growth level at 12 h, *E. coli* K12 multiplied faster before 8 h.

In the presence of plantaricin BM-1, wild-type *E. coli* K12 grew slowly over the 12 h period, and the concentration of viable bacteria cells at 12 h was found to be 7.9 log_10_ CFU/mL, indicating that wild-type *E. coli* K12 was moderately sensitive to plantaricin BM-1. In contrast, *E. coli* JW4073 and *E. coli* JW4074 grew slowly from 0 to 4 h, with almost no increase in the number of viable bacterial cells. The number of viable *E. coli* JW4073 and *E. coli* JW4074 bacterial cells at 12 h were 6.4 log_10_ CFU/mL and 5.8 log_10_ CFU/mL, respectively, and these values were significantly (*P* < 0.05) lower than that obtained for wild-type *E. coli* K12. Our results indicated that mutations in *BasS* and *BasR* increased the sensitivity of *E. coli* K12 to plantaricin BM-1.

In the presence of plantaricin BM-1, the growth curves of the supplementary strains (*E. coli* ReJW4073 and *E. coli* ReJW4074) were similar to that of wild-type *E. coli* K12, indicating that the sensitivity of the supplementary strains increased to the same level as that of the wild-type strain. This further indicated that the BasS/BasR TCS regulates the sensitivity of *E. coli* to plantaricin BM-1.

### Proteomic Analysis

**FIGURE 3:**
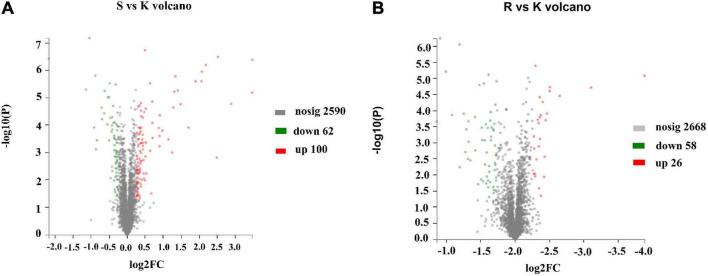
Changes in the *E. coli* K12 proteome following *BasS* and *BasR* deletion. The volcano plots of the 2752 identified proteins are shown. **(A)** Represents the *E. coli* JW4073 (S)/*E. coli* K12 (K) group, while **(B)** represents the *E. coli* JW4074 (R)/*E. coli* K12 (K) group. The different colors indicate different fold changes. Red represents proteins with fold change values greater than 1.20, while green represents proteins with the fold change values less than 0.83 (*P* < 0.05).

To determine the mechanism by which the BasS/BasR TCS affects the sensitivity of *E. coli* K12 to plantaricin BM-1, a TMT proteomic analysis was performed on *E. coli* K12, *E. coli* JW4073, and *E. coli* JW4074. A total of 2,752 proteins were identified, with a 1.2-fold change in threshold differential expression (upregulated Fold change >1.2, downregulated Fold change <0.83), and a statistically tested Student’s *t*-test *P*-value < 0.05 considered as the significance threshold for biological information in the differential protein scientific analysis. As shown in Figure 3A, the expression levels of 162 of these proteins changed in the *E. coli* JW4073/*E. coli* K12 comparison group, i.e., 100 upregulated and 62 downregulated proteins (*p* < 0.05). As shown in Figure 3B, in the *E. coli* JW4074/*E. coli* K12 comparison group, the expression levels of 84 proteins changed, i.e., 26 upregulated and 58 downregulated proteins (*p* < 0.05).

Based on the analysis of the subcellular location of the differentially expressed proteins in the two comparison groups, we found that proteins which showed significant changes in their expression levels were mainly concentrated in the cytoplasm in both *E. coli* JW4073 and *E. coli* JW4074 strains, accounting for 87.65 and 95.64% of the total differential proteins, respectively. The other proteins were distributed in the plasma membrane and the extracellular milieu. There were relatively greater changes in plasma membrane protein levels in *E. coli* JW4074 than in *E. coli* JW4073, accounting for 10.49% of all differentially expressed proteins (Data not shown).

Gene ontology (GO) describes the role of eukaryotic genes and proteins in cells through the establishment of a controlled vocabulary set in a dynamic form, so as to fully describe the properties of genes and gene products in organisms. KEGG functional annotation analysis was performed on the proteins to verify the functional classification of the pathway or the role of the protein at the functional level. Fisher’s exact test was used to compare categorical data. At corrected *P*-values (P adjust) < 0.05, the KEGG pathway function was considered to be significantly enriched.

**FIGURE 4:**
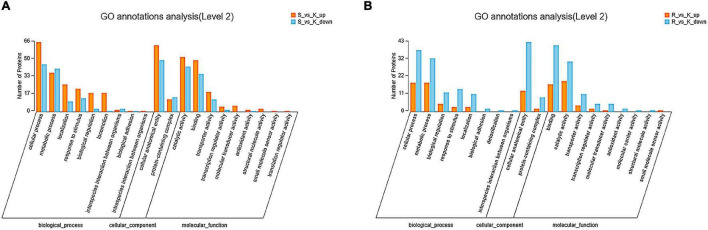
Gene ontology annotation classification map of differentially expressed proteins in the *E. coli* JW4073(S)/*E. coli* K12(K) **(A)** and *E. coli* JW4074(R)/*E. coli* K12(K) groups **(B)**.

In the *E. coli* JW4073/*E. coli* K12 group, among the 162 differentially expressed proteins, 131 were labeled as biological process (BP), and their content in relation to cellular and metabolic process-related proteins were 50.38 and 28.24%, respectively (Figure 4A). In the *E. coli* JW4074/*E. coli* K12 group, among the 84 differentially expressed proteins, 73 proteins were labeled as biological process (BP), and their content in relation to cellular and metabolic process-related proteins were 76.71 and 69.86%, respectively (Figure 4B).

**FIGURE 5:**
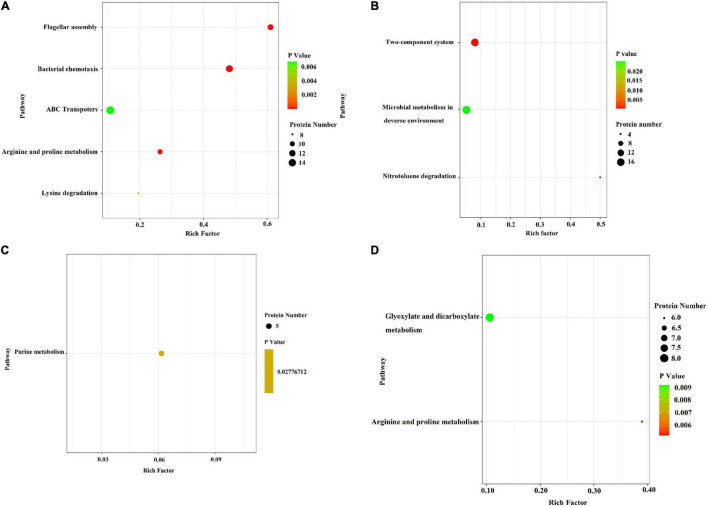
Bubble chart of differentially expressed protein-related KEGG enrichment pathways in the *E. coli* JW4073(S)/*E. coli* K12(K) group and *E. coli* JW4074(R)/*E. coli* K12(K) group. **(A,B)** Represent differentially expressed protein enrichment pathways in the *E. coli* JW4073(S)/*E. coli* K12(K) group; **(C,D)** represent differentially expressed protein enrichment pathways in the *E. coli* JW4074(R)/*E. coli* K12(K) group, *P* < 0.05.

In the *E. coli* JW4073/*E. coli* K12 group, upregulated proteins were mainly involved in five pathways, i.e., the flagella assembly (eco02040) pathway, the bacterial chemotaxis pathway (eco02030), the arginine and proline metabolic pathway (eco00330), the lysine degradation pathway (eco00310), and the ABC transport pathway (eco02010) (Figure 5A). The downregulated protein enrichment pathways included the TCS pathway (eco02020), the nitrotoluene degradation pathway (eco00633), and microbial metabolism in different environments (eco01120) (Figure 5B). In the *E. coli* JW4074/*E. coli* K12 group, only the purine metabolic pathway (eco00230) was significantly enriched in the 26 upregulated proteins (Figure 5C). The arginine and proline metabolic pathways (eco00330), as well as the glyoxylate and dicarboxylate metabolic pathways (eco00660), were significantly enriched in 58 downregulated proteins (Figure 5D).

As compared to the wild-type *E. coli* K12 strain, in *E. coli* JW4073 and *E. coli* JW4074 strains, there were 15 proteins with the same differential expression trend, including 1 up-regulated protein and 14 down-regulated proteins (Table 3). These proteins were found to be mainly involved in biological processes and cellular component and molecular functions. The only upregulated protein common to this protein was YmgA, which is a TCS-related protein. As concerns the 14 downregulated proteins, the glycerophospholipid metabolic pathway (eco00564), in which three different proteins (GlpA, GlpB, and GlpC) are involved, was significantly enriched. The expression levels of GlpA, GlpB, and GlpC were downregulated 1. 54-, 1. 41-, and 1.64-folds, respectively, in *E. coli* JW4073, and 1. 75-, 1. 50-, and 2.01-folds, respectively, in *E. coli* JW4074. These proteins are important components of the simple ATP-generation electron transport pathway used by *E. coli* during growth in anaerobic conditions. Of the 14 downregulated proteins, 8 proteins, including pyruvate formate lyase (PflD), citrate lyase (CitF), dicarboxylate transporters (DcuB, DcuC), fumarate synthase (FumA), and anaerobic glycerol-3-phosphate dehydrogenase (GlpABC), were found to be related to the TCA cycle (Table 3).

**TABLE 3:** The BasS/BasR TCS regulates changes in protein expression in *E. coli* JW4073 and *E. coli* JW4074.

Accession number	Protein	Description	Fold change	
			*E. coli* JW4073/*E. coli* K12	*E. coli* JW4074/*E. coli* K12
b0615	CitF	Citrate lyase subunit alpha	0.823941	0.751429
b0621	DcuC	Anaerobic C4-dicarboxylate transporter DcuC	0.725937	0.751489
b0873	hcp	Hydroxylamine reductase	0.70245	0.827488
b0990	CspG	Cold shock protein CspG	0.816885	0.668902
b2241	GlpA	Anaerobic glycerol-3-phosphate dehydrogenase subunit A	0.64914	0.571328
b2242	GlpB	Anaerobic glycerol-3-phosphate dehydrogenase subunit B	0.707122	0.667276
b2243	GlpC	Anaerobic glycerol-3-phosphate dehydrogenase subunit C	0.611355	0.498009
b3829	MetE	5-methyltetrahydropteroyltriglutamate–homocysteine S-methyltransferase	0.829016	0.729757
b3113	RidA	Enamine/imine deaminase	0.803088	0.800797
b3114	PflD	2-ketobutyrate formate-lyase/pyruvate formate-lyase	0.713731	0.764876
b4122	FumA	Class I fumarate hydratase	0.688427	0.77814
b4123	DcuB	Anaerobic C4-dicarboxylate transporter DcuB	0.642033	0.783913
b4131	CadA	Lysine decarboxylase CadA	0.542167	0.469261
b4430		Hypothetical protein, partial	0.54793	0.60629

### RT-qPCR

**FIGURE 6:**
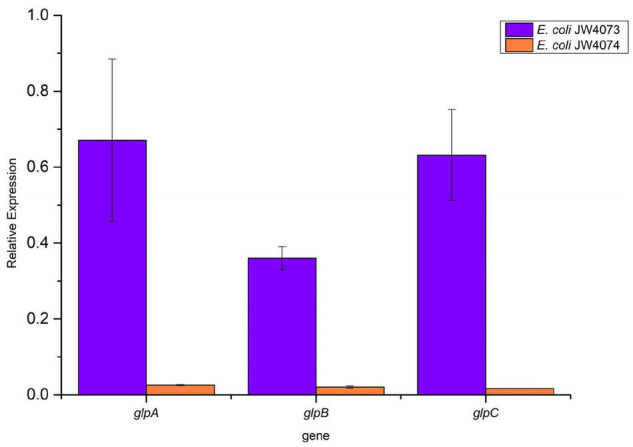
Expression levels of *glpA*, *glpB*, and *glpC* in *E. coli* JW4073 and *E. coli* JW4074.

RT-qPCR findings (Figure 6) showed a significant decrease in the transcriptional levels of *glpA*, *glpB*, and *glpC* in mutant strains (*p* < 0.05). Their expression levels were downregulated 0.67-, 0.36-, and 0.63-folds, respectively, in *E. coli* JW4073, and downregulated 0.03-, 0.02-, and 0.02-folds, respectively, in *E. coli* JW4074. These results were consistent with the findings of the proteomic analysis.

### Effects of the Downregulation of Tricarboxylic Acid-Related Proteins on the Sensitivity of *Escherichia coli* to BM-1

**FIGURE 7:**
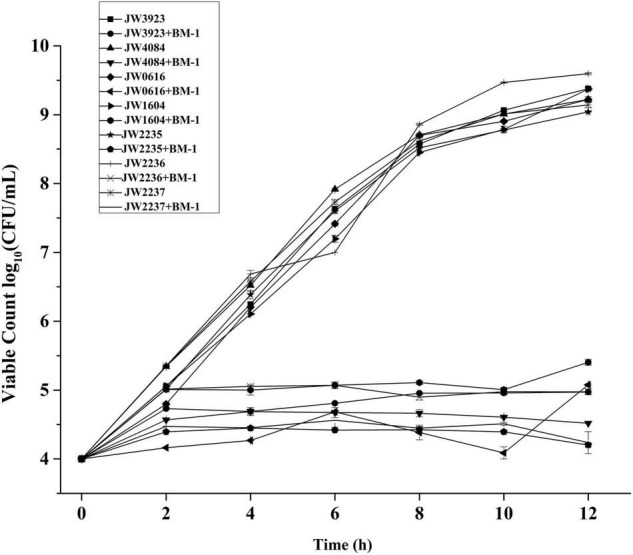
Effects of plantaricin BM-1 on the growth of mutant *E. coli* JW3923(*pflD*), *E. coli* JW4084(*dcuB*), *E. coli* JW0616(*dcuC*), *E. coli* JW1604(*fumA*), *E. coli* JW2235(*glpA*), *E. coli* JW2236(*glpB*), and *E. coli* JW2237(*glpC*) strains.

The effects of plantaricin BM-1 on the growth of *pflD*, *dcuB*, *dcuC*, *fumA*, *glpA*, *glpB*, and *glpC E. coli* mutants were assessed by generating standard growth curves (Figure 7). In the absence of plantaricin BM-1, all the mutant strains showed similar exponential growth. Related gene mutations did not affect the normal growth ability of *E. coli*. In the presence of plantaricin BM-1, mutant *E. coli* strains grew slowly over the 12 h period, with approximately no increase in the number of viable bacterial cells, and their counts were significantly (*P* < 0.05) lower than that of the wild-type *E. coli* K12 strain. We found that the BasS/BasR TCS affected the sensitivity of *E. coli* to plantaricin BM-1 by regulating *PflD*, *DcuB*, *DcuC*, *FumA*, *GlpA*, *GlpB*, and *GlpC* expression.

## Discussion

The BasS/BasR TCS is a transcription regulatory system induced by iron and zinc; it is mainly involved in the regulation of reactions between divalent metal ions (Fe, Zn) and genes that respond to acidic and anaerobic growth conditions. Following the stimulation of the BasS protein by external signals, the histidine residue in its hisKA domain is phosphorylated and sends a signal to BasR for its activation. Then, the BasR protein further regulates other target genes in response to external stimuli (Gunn, 2008; Yu et al., 2015). The target genes activated by BasR can be divided into three groups. The first group includes genes involved in membrane structure formation and modification; the second group includes regulatory membrane functional genes; and the third group includes genes involved in cell response to stress (Ogasawara et al., 2012). In a previous study, Yu et al. (2020) constructed a *basSR*-deficient mutant strain (XY1) using an APECX40 (WT) parent strain, and performed high-throughput sequencing on both strains to determine their transcriptional profiles. They found that *basSR* deletion induced a downregulation of the transcript levels of a series of biofilm- and virulence-related genes. The findings of biofilm formation assays and animal model experiments showed that *BasSR* deletion induced the inhibition of biofilm formation *in vitro* and decreased bacterial virulence and colonization *in vivo*. In addition, electrophoretic mobility shift assays confirmed that the BasR protein could bind to the promoter regions of several biofilm- and virulence-related genes, including *ais*, *opgC*, and *fepA*. The findings of this study suggest that the BasS/BasR TCS might play the role of a global regulator during the pathogenesis of APEC infections (Yu et al., 2020). Furthermore, in a previous study, we found that YbfA, a DUF2517 domain-containing protein, null mutation decreased the sensitivity of *E. coli* K12 to plantaricin BM-1 (Chen et al., 2021). Electron microscopy showed that the ybfA null mutation induced a decrease in plantaricin BM-1-induced surface rupture and contraction, and mitigated the effects of plantaricin BM-1 on *E. coli* K12 cell membrane morphology. Proteomic analysis showed that ybfA mutation induced an upregulation of some outer membrane and plasma membrane proteins in *E. coli* K12. Moreover, we found that the expression levels of BasS/BasR TCS-related proteins significantly increased (*P* < 0.05). In addition, there was a significant increase in the expression levels of TCS-regulated downstream proteins, including DgkA, FliC, and MlaE, which are involved in the regulation of cell membrane structure and function. Growth curve analysis showed that the absence of the BasS/BasR TCS induced a significant increase in the sensitivity of *E. coli* K12 to plantaricin BM-1. Therefore, the BasS/BasR TCS may be actively involved in the regulation of cell membrane structure and function, thereby reducing bacterial sensitivity to plantaricin BM-1. However, so far, the mechanism by which the BasS/BasR TCS affects the sensitivity of *E. coli* to plantaricin BM-1 has not been fully elucidated.

In this study, by comparing the proteomic data of the two comparison groups, we found that proteins which showed significant changes in their expression levels were mainly concentrated in the cytoplasm in both *E. coli* JW4073 and *E. coli* JW4074 strains, while the other proteins were distributed in the plasma membrane and extracellular milieu. In addition, there were relatively greater changes in the levels of plasma membrane proteins in *E. coli* JW4073 strains than in *E. coli* JW4074 strains. These proteins are mainly involved in bacterial biological processes and material metabolism, including amino acid and lipid synthesis and metabolism, as well as bacterial chemotaxis. Moreover, proteomic data showed that as compared to the wild-type *E. coli* K12 strain, in *E. coli* JW4073 and *E. coli* JW4074 strains, 15 proteins, i.e., 1 upregulated protein and 14 downregulated proteins, exhibited the same differential expression trend. These proteins were found to be mainly involved in biological processes and cellular component and molecular functions. Among the 14 down regulated proteins, 8 (PflD, CitF, DcuB, DcuC, FumA, GlpA, GlpB, and GlpC) were found to be related to the TCA cycle. All of these proteins have different molecular functions and participate in different molecular pathways. Moreover, the deletion of the genes encoding PflD, DcuB, DcuC, FumA, GlpA, GlpB, and GlpC was found to induce a significant increase the sensitivity of *E. coli* to plantaricin BM-1.

The TCA cycle is a key component of the energy production metabolic pathway of all aerobic organisms (Akram, 2014). Some antibacterial substances inhibit the normal bacterial metabolic pathway by inhibiting the TCA cycle and glycolysis, as well as by inducing intracellular metabolic imbalance. Chlorogenic acid (CGA) induces a significant decrease in intracellular adenosine triphosphate (ATP) concentrations, possibly by regulating substance and energy metabolism or cell signal transduction. CGA-induced stress exerts bacteriostatic effects by inducing intracellular metabolic imbalance in the TCA cycle and glycolysis (Wu et al., 2020). Previous studies have shown that the plantaricin BM-1-induced stress response in *E. coli* K12 involves multiple pathways (Wang et al., 2020). *E. coli* K12 can upregulate peptidoglycan synthesis and the TCA cycle, and maintain its ultrastructural integrity by regulating membrane protein expression, thereby resisting cationic peptide-induced damage (Wang et al., 2020).

Pyruvate formate lyase (PflD) can catalyze the decomposition of pyruvate into formic acid and its subsequent conversion into CO_2_ and H_2_ (Choi et al., 2013). It was found to be downregulated 0.71- and 0.76-folds in the *basS* and *basR* mutants, respectively. A decrease in PflD expression can affect normal pyruvate metabolism and cause pyruvate accumulation (Becker et al., 2013). DcuB and DcuC are the main succinate efflux transporters, and function as independent and mutually redundant succinate efflux transporters. The absence of DcuB and DcuC can significantly affect succinic acid yield (Chen et al., 2014). In addition, FumA, which is involved in the fumarate metabolic pathway and functions as a receptor in the electron transport chain, was found to be downregulated 0.69- and 0.78-folds in the *basS* mutant and *basR* mutant, respectively. It was found to affect the citric acid cycle by regulating fumaric acid synthesis, thereby inhibiting the TCA cycle. These findings are similar to those reported by Miao et al. (2015), who found that F1 could downregulate FamB, thereby inhibiting *E. coli* growth (Miao et al., 2015). FUMA and FUMB, which are class I fumarases in *E. coli*, are its main aerobic and anaerobic enzymes. Their high affinities for fumarate and malate indicate that they are specifically adapted to play reciprocal roles in the overall fumarate oxidation process *via* the citric acid cycle (FUMA) and in the ultimate reduction of malate by providing fumarate, which serves as an anaerobic electron acceptor (FUMB) (Woods et al., 1988). In this study, the effects of the proteins, PflD, DcuB, DcuC, and FumA, on the sensitivity of *E. coli* to plantaricin BM-1 were similar to those reported by Matsumoto et al. (2020). They found that *Staphylococcus aureus* regulates the TCA cycle by producing pyruvate and reducing its tolerance to betamethasone valerate (BV). Inhibiting the activity of succinate dehydrogenase was found to increase the sensitivity of *S. aureus* to BV (Matsumoto et al., 2020).

In addition, the glycerol phospholipid metabolic pathway which involves GlpABC was found to transfer its reduced equivalent from sn-glycerol-3-phosphate (G-3-P) to a short electron transfer chain that ends with fumaric acid as the final electron acceptor (Cole et al., 1988). The GlpAB dimer carries the catalytic site for G-3-P oxidation, and its active site, which binds to the domain, may be located in the GlpB subunit. The GlpC subunit contains two typical cysteine clusters of iron-sulfur rotation domains. This subunit is closely related to the bacterial envelope, and may serve as the membrane anchor for the GlpAB dimer (Varga and Weiner, 1995). The expression levels of these three proteins were found to be simultaneously downregulated in BasS/BasR TCS mutants, and these findings were consistent with those of the RT-qPCR experiments. In *E. coli*, fumaric acid serves in the electron transfer chain as the electron transfer receptor that connects the TCA cycle. Citric acid lyase (CitF), which is a key enzyme in the citric acid cycle and cooperates with FumA, was found to be downregulated 0.82- and 0.75-folds, respectively, in *basS* mutant and *basR* mutant. This reduction in its expression significantly affected fumaric acid synthesis, thereby affecting energy transfer in *E. coli*, and consequently affecting its growth and multiplication.

In summary, decreased BasS/BasR TCS expression quantity can increase the sensitivity of wild-type *E. coli* K12 to plantaricin BM-1. Following *basS/R* gene knockout, there was a significant change in the expression levels of several cellular cytoplasmic metabolic pathway-related proteins. Mutations in the BasS/BasR TCS induced significant changes in cellular pathways, including the TCA and glycerophospholipid metabolic pathways, and also induced changes in the regulation of bacterial metabolism and energy production, which consequently affected the sensitivity of *E. coli* to plantaricin BM-1.

## Data Availability Statement

The datasets presented in this study can be found in online repositories. The names of the repository/repositories and accession number(s) can be found below: https://iprox.cn/, IPX0003932000.

## Author Contributions

YL conceived and designed the experiments, performed the experiments, and analyzed the data. YW and XC analyzed the data. JJ, HL, and YH contributed materials. HZ conceptualization and methodology. YX conceived and designed the experiments. All authors contributed to the manuscript and approved the submitted version.

## Conflict of Interest

The authors declare that the research was conducted in the absence of any commercial or financial relationships that could be construed as a potential conflict of interest.

## Publisher’s Note

All claims expressed in this article are solely those of the authors and do not necessarily represent those of their affiliated organizations, or those of the publisher, the editors and the reviewers. Any product that may be evaluated in this article, or claim that may be made by its manufacturer, is not guaranteed or endorsed by the publisher.

## Footnotes

1
http://www.genome.jp/kegg/

## References

[B1] Akram M. (2014). Citric acid cycle and role of its intermediates in metabolism. *Cell Biochem. Biophys.* 68 475–478. 10.1007/s12013-013-9750-1 24068518

[B2] Becker J. Reinefeld J. Stellmacher R. Schäfer R. Lange A. Meyer H. (2013). Systems-wide analysis and engineering of metabolic pathway fluxes in bio-succinate producing Basfia succiniciproducens. *Biotechnol. Bioeng.* 110 3013–3023. 10.1002/bit.24963 23832568

[B3] Chen J. Zhu X. Tan Z. Xu H. Tang J. Xiao D. (2014). Activating C4-dicarboxylate transporters DcuB and DcuC for improving succinate production. *Appl. Microbiol. Biotechnol.* 98 2197–2205. 10.1007/s00253-013-5387-7 24323285

[B4] Chen X. Liu Y. Jin J. Liu H. Hao Y. Zhang H. (2021). YbfA Regulates the Sensitivity of *Escherichia coli* K12 to Plantaricin BM-1 viathe BasS/BasR Two-Component Regulatory System. *Front. Microbiol.* 12:659198. 10.3389/fmicb.2021.659198 34484135 PMC8415914

[B5] Choi S. Kim H. U. Kim T. Y. Kim W. J. Lee M. H. Lee S. Y. (2013). Production of 4-hydroxybutyric acid by metabolically engineered Mannheimia succiniciproducens and its conversion to γ-butyrolactone by acid treatment. *Metab. Eng.* 20 73–83. 10.1016/j.ymben.2013.09.001 24055777

[B6] Cole S. T. Eiglmeier K. Ahmed S. Honore N. Elmes L. Anderson W. F. (1988). Nucleotide sequence and gene-polypeptide relationships of the glpABC operon encoding the anaerobic sn-glycerol-3-phosphate dehydrogenase of *Escherichia coli* K-12. *J. Bacteriol.* 170 2448–2456. 10.1128/jb.170.6.2448-2456.1988 3286606 PMC211154

[B7] Cotter P. D. Hill C. Ross R. P. (2005). Bacteriocins: developing innate immunity for food. *Nat. Rev. Microbiol.* 3 777–788. 10.1038/nrmicro1273 16205711

[B8] Cui Y. Zhang C. Wang Y. Shi J. Zhang L. Ding Z. (2012). Class IIa bacteriocins: diversity and new developments. *Int. J. Mol. Sci.* 13 16668–16707. 10.3390/ijms131216668 23222636 PMC3546714

[B9] Diep D. B. Skaugen M. Salehian Z. Holo H. Nes I. F. (2007). Common mechanisms of target cell recognition and immunity for class II bacteriocins. *Proc. Natl. Acad. Sci. U S A* 104 2384–2389. 10.1073/pnas.0608775104 17284603 PMC1892938

[B10] Gunn J. S. (2008). The *Salmonella* PmrAB regulon: Lipopolysaccharide modifications, antimicrobial peptide resistance and more. *Trends Microbiol.* 16 264–290. 10.1016/j.tim.2008.03.007 18467098

[B11] Hagiwara D. Yamashino T. Mizuno T. (2004). A genome-wide view of the *Escherichia coli* BasS–BasR two-component system implicated in iron-responses. *Biosci. Biotechnol. Biochem.* 68 1758–1767. 10.1271/bbb.68.1758 15322361

[B12] Hassan M. Kjos M. Nes I. F. Diep D. B. Lotfipour F. (2012). Natural antimicrobial peptides from bacteria: characteristics and potential applications to fight against antibiotic resistance. *J. Appl. Microbiol.* 113 723–736. 10.1111/j.1365-2672.2012.05338.x 22583565

[B13] Juhas M. Ajioka J. W. (2016). Integrative bacterial artificial chromosomes for DNA integration into the Bacillus subtilis chromosome. *J. Microbiol. Methods* 125 1–7. 10.1016/j.mimet.2016.03.017 27033694

[B14] Kjos M. Borrero J. Opsata M. Birri D. J. Holo H. Cintas L. M. (2011). Target recognition, resistance, immunity and genome mining of class II bacteriocins from Gram-positive bacteria. *Microbiology* 157 3256–3267. 10.1099/mic.0.052571-0 21980118

[B15] Kumariya R. Garsa A. K. Rajput Y. S. Sood S. K. Akhtar N. Patel S. (2019). Bacteriocins: Classification, synthesis, mechanism of action and resistance development in food spoilage causing bacteria. *Microb. Pathogenesis* 128 171–177. 10.1016/j.micpath.2019.01.002 30610901

[B16] Lee L. J. Barrett J. A. Poole R. K. (2005). Genome-wide transcriptional response of chemostat-cultured *Escherichia coli* to zinc. *J. Bacteriol.* 187 1124–1134. 10.1128/JB.187.3.1124-1134.2005 15659689 PMC545701

[B17] Matsumoto Y. Nakashima T. Cho O. Ohkubo T. Kato J. Sugita T. (2020). Pyruvate-triggered TCA cycle regulation in Staphylococcus aureus promotes tolerance to betamethasone valerate. *Biochem. Biophys. Res. Comm.* 528 318–321. 10.1016/j.bbrc.2020.05.035 32475641

[B18] Miao J. Chen F. Duan S. Gao X. Liu G. Chen Y. (2015). iTRAQ-Based Quantitative Proteomic Analysis of the Antimicrobial Mechanism of Peptide F1 against *Escherichia coli*. *J. Agricult. food chem.* 63 7190–7197. 10.1021/acs.jafc.5b00678 26208148

[B19] Mizuno T. Aiba H. Oshima T. Mori H. Wanner B. L. (2003). “Genome-wide analysis of *Escherichia coli* histidine kinases,” in *“Histidine Kinases in Signal Transduction”*, eds Inouye M. Dutta R. (New York: Academic Press), 192–202.

[B20] Nagasawa S. Ishige K. Mizuno T. (1993). Novel members of the two-component signal transduction genes in *Escherichia coli*. *J. Biochem.* 114 350–357. 10.1093/oxfordjournals.jbchem.a124180 8282725

[B21] Ogasawara H. Shinohara S. Yamamoto K. Ishihama A. (2012). Novel regulation targets of the metal-response BasS-BasR two-component system of *Escherichia coli*. *Microbiology* 158(Pt 6), 1482–1492. 10.1099/mic.0.057745-0 22442305

[B22] Rodríguez J. M. Martínez M. I. Kok J. (2002). Pediocin PA-1, a wide spectrum bacteriocin from lactic acid bacteria. *Crit. Rev. Food Sci. Nutr.* 42 91–121. 10.1080/10408690290825475 11934133

[B23] Tomoya B. Takeshi A. Miki H. Takai Y. Okumura Y. Baba M. (2006). Construction of *Escherichia coli* K-12 in-frame, single-gene knockout mutants: the Keio collection. *Mol. Syst. Biol.* 2:2006.0008. 10.1038/msb4100050 16738554 PMC1681482

[B24] Telhig S. Ben Said L. Zirah S. Fliss I. Rebuffat S. (2020). Bacteriocins to Thwart Bacterial Resistance in Gram Negative Bacteria. *Front. Microbiol.* 11:586433. 10.3389/fmicb.2020.586433 33240239 PMC7680869

[B25] Varga M. E. Weiner J. H. (1995). Physiological role of GlpB of anaerobic glycerol-3-phosphate dehydrogenase of *Escherichia coli*. *Biochem. cell* 73 147–153. 10.1139/o95-018 7576488

[B26] Wang H. Xie Y. Zhang H. Jin J. Zhang H. (2020). Quantitative proteomic analysis reveals the influence of plantaricin BM-1 on metabolic pathways and peptidoglycan synthesis in *Escherichia coli* K12. *PloS one* 15:e0231975. 10.1371/journal.pone.0231975 32324803 PMC7179913

[B27] West A. H. Stock A. M. (2001). Histidine kinases and response regulator proteins in two-component signaling systems. *Trends Biochem. Sci.* 26 369–376. 10.1016/s0968-0004(01)01852-7 11406410

[B28] Woods S. A. Schwartzbach S. D. Guest J. R. (1988). Two biochemically distinct classes of fumarase in *Escherichia coli*. *Biochim. et Biophys. Acta* 954 14–26. 10.1016/0167-4838(88)90050-7 3282546

[B29] Wu Y. Liang S. Zhang M. Wang Z. Wang Z. Ren X. (2020). The Effect of Chlorogenic Acid on Bacillus subtilis Based on Metabolomics. *Molecules* 25:4038. 10.3390/molecules25184038 32899667 PMC7571229

[B30] Yu L. Wang H. Han X. Li W. Xue M. Qi K. (2020). The two-component system, BasSR, is involved in the regulation of biofilm and virulence in avian pathogenic *Escherichia coli*. *Avian Pathol.* 49 532–546. 10.1080/03079457.2020.1781791 32894030

[B31] Yu Z. L. Qin W. R. Lin J. X. Fang S. Qiu J. (2015). Antibacterial mechanisms of polymyxin and bacterial resistance. *Biomed. Res. Int.* 2015:679109. 10.1155/2015/679109 25664322 PMC4312571

[B32] Zhang H. Liu L. Hao Y. Zhong S. Liu H. Han T. (2013). Isolation and partial characterization of a bacteriocin produced by Lactobacillus plantarum BM-1 isolated from a traditionally fermented Chinese meat product. *Microbiol. Immunol.* 57 746–755. 10.1111/1348-0421.12091 24033418

